# Integrative Study of Dipsaci Radix and Phlomidis Radix: Nomenclature, Morphology, DNA-Based Authentication, and Comparative Effects on Osteoclastogenesis

**DOI:** 10.3390/ph18091418

**Published:** 2025-09-20

**Authors:** Jun-Ho Song, Yun-Soo Seo, Yeseul Kim, Sohee Jeong, Sungyu Yang, Goya Choi, Joong-Sun Kim, Inkyu Park

**Affiliations:** 1 Department of Biology, Chungbuk National University, Cheongju 28644, Republic of Korea; jhsong@chungbuk.ac.kr; 2 Herbal Medicine Resources Research Center, Korea Institute of Oriental Medicine, Naju 58245, Republic of Korea; sys0109@kiom.re.kr (Y.-S.S.); sgyang81@kiom.re.kr (S.Y.); serparas@kiom.re.kr (G.C.); 3 Department of Biology, Changwon National University, Changwon 51140, Republic of Korea; 20257520@gs.cwnu.ac.kr; 4 College of Veterinary Medicine and BK21 FOUR Program, Chonnam National University, Gwangju 61186, Republic of Korea; sohee0460@naver.com

**Keywords:** Dipsaci Radix, Phlomidis Radix, nomenclature, *Dipsacus japonicus*, DNA barcoding, osteoblasts, bone resorption

## Abstract

**Background/Objectives**: Dipsaci Radix (*Dipsacus asper*) and Phlomidis Radix (*Phlomoides umbrosa*) are both traditional medicines used in Korea and China for various bone-associated diseases. However, the two are misused due to similarities in name and appearance. Additionally, *D. japonicus* root frequently contaminates Dipsaci Radix in Korean herbal markets. **Methods**: We examined morphological plant traits and performed a DNA barcoding analysis using ITS2 and *matK* sequences to differentiate between these three species. The effects of root extracts on bone resorption and osteoclast differentiation, measured as tartrate-resistant acid phosphatase (TRAP)-positive cell formation, were evaluated using mouse (5 weeks male ICR mice) bone marrow-derived macrophages. Cytotoxicity assays were conducted to assess extract safety. **Results**: *Phlomoides umbrosa* is easily distinguished by its verticillaster inflorescences and 2-labiate corollas. *Dipsacus asper* and *D. japonicus*, which share globose inflorescences, are distinguishable by flower color and leaf lobation. The ITS2 and *matK* sequences clearly differentiated the three species, with haplotype analysis supporting their genetic distinctiveness, enabling robust species discrimination. All three extracts decreased osteoclastic bone resorption and inhibited TRAP-positive cell formations in a dose-dependent manner. Only the *D. japonicus* extract demonstrated toxicity. **Conclusions**: This integrative study provides the current scientific names of the original species and proposes their use in the Korean Herbal Pharmacopoeia. Moreover, a reasonable molecular method for authenticating medicinal materials is suggested. *Dipsacus japonicus* shows promise as an additional origin species in the Korean Pharmacopoeia. However, processing methods that reduce toxicity must be discovered.

## 1. Introduction

Medicinal plants and plant-derived medications are widely used as traditional medicines and natural substitutes or supplements for synthetic drugs [1]. However, adulteration and contamination with plants of other genera and related species are frequent [2]. Moreover, the misuse of medicines due to the similarity of plants’ common or pharmaceutical names or other nomenclature issues is especially common [3]. Thus, it is essential to accurately morphologically identify and describe authentic and original plant species. Moreover, accessible and reliable techniques for checking the quality of herbal products and discriminating pure materials from adulterated or contaminated materials are needed.

The roots of *Dipsacus asper* Wall. ex DC. (synonym *D. asperoides* C. Y. Cheng & T. M. Ai) (Caprifoliaceae, formerly Dipsacaceae) and *Phlomoides umbrosa* (Turcz.) Kamelin & Makhm. (synonym *Phlomis umbrosa* Turcz.) (Lamiaceae = Labiatae) have been used as important traditional medicines in China and Korea. *Dipsacus asper* is commonly known as Dipsaci Radix but also has the name “Xu-Duan” in the Chinese Pharmacopoeia (CP) and the herbal medicine name “Sok-Dan” in the Korean Herbal Pharmacopoeia (KHP) [4]. However, *P. umbrosa*, known as Phlomidis Radix, has a similar herbal medicine name, “Han-Sok-Dan”, in the KHP and the same botanical name “Sok-Dan” in the Flora of Korea. Unfortunately, adulteration and contamination are persistent problems in the Dipsaci Radix distributed in Korean herbal markets. The roots of *D. japonicus* Miq., a species closely related to *D. asper*, are mixed and distributed with those of *D. asper* because of their morphological similarities. Similarly, the roots of *P. umbrosa*, whose pharmacological effects differ from those of *D. asper*, are also commonly mixed with those of Dipsaci Radix because of the similarity of their common names [5].

DNA barcoding is an effective tool for species identification that does not rely on morphological characteristics, enabling rapid and accurate species differentiation [6]. This method targets specific genetic loci, using them to distinguish organisms based on subtle nucleotide variations. It utilizes short DNA fragments to enhance analytical efficiency [7]. In plants, DNA barcoding primarily utilizes regions from the chloroplast and nuclear genomes, including chloroplast genome fragments like *rbcL*, *matK*, and *psbA*-*trnH*, as well as the internal transcribed spacer (ITS) nuclear gene region [8]. Notably, the second internal transcribed spacer (ITS2) has been proposed as a standard DNA barcode for identifying species closely related to medicinal plants [9]. Several studies have successfully used DNA sequencing to distinguish between closely related species, not only across different genera but also within the same genus [10,11,12]. In particular, ITS2 has been shown to be highly effective for the identification of Dipsaci Radix [13]. Furthermore, species-specific SCAR markers based on indels in the *accD* and *matK* regions have been developed to discriminate Dipsaci Radix from Phlomidis Radix, and their applicability for the accurate identification of these species has been demonstrated [14].

Dipsaci Radix, the dried root of *D. asper*, was first recorded in the *Shennong’s Classic of Materia Medica*, where it was noted for its ability to “renew fractures and join bones” [15]. It has traditionally been used to treat musculoskeletal disorders, and modern studies have demonstrated its efficacy in promoting osteoblast proliferation, preventing bone injury, alleviating osteoporosis, and exerting anti-inflammatory effects [16,17,18]. Phlomidis Radix has also been reported to enhance bone mineralization in osteoblasts and to positively influence osteogenesis [19] and has been confirmed to effectively inhibit the differentiation and bone resorption of osteoclasts [20]. Phlomidis Radix increased bone density in mouse and rat osteoporosis models and, based on various indices, alleviated osteoporosis symptoms [21,22]. However, no studies comparing the effects of Dipsaci Radix and Phlomidis Radix on bone formation have been conducted.

In the past, studies have mixed results from Dipsaci Radix and Phlomidis Radix or lumped them together under a single name, causing confusion [20]. Although previous studies have applied morphology, DNA barcoding, or osteoclast assays separately, our work uniquely integrates these approaches across *D. asper*, *D. japonicus*, and *P. umbrosa* [13,14,16,17,18,23]. This allows simultaneous assessment of authentication markers and pharmacological potential, establishing *D. japonicus* as a candidate for pharmacopoeial standardization. The novelty of this study is therefore the combined methodological framework and the comparative evaluation across multiple species, which has not been previously reported.

In this study, we described the detailed morphological characteristics of *D. asper*, *D. japonicus*, and *P. umbrosa* plants to facilitate herbal medicine authentication. We also analyzed the ability of universal DNA barcodes to distinguish between *D. asper*, *D. japonicus*, and *P. umbrosa* and explored effective authentication strategies. Furthermore, we examined the effects of not only Dipsaci Radix and Phlomidis Radix but also *D. japonicus* on osteoclast differentiation to confirm whether *D. japonicus* can be added to the Pharmacopoeia.

## 2. Results

### 2.1. Morphological Characteristics of D. asper, D. japonicus, and P. umbrosa

**Figure 1:**
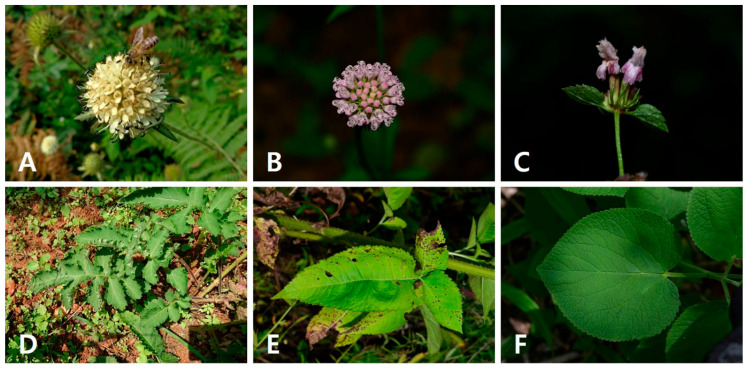
Inflorescence (**A**–**C**) and leaf (**D**–**F**) morphologies of *Dipsacus asper* (**A**,**D**), *D. japonicus* (**B**,**E**), and *Phlomoides umbrosa* (**C**,**F**).

*Dipsacus asper* and *D. japonicus* are morphologically similar, sharing inflorescence, corolla, and fruit characteristics, because they are grouped in the same family (Caprifoliaceae, formerly Dipsacaceae). However, *P. umbrosa* is easily distinguishable from the other two species based on the morphology of the aerial parts, as this species is from a different family (Lamiaceae, Labiatae) (Figure 1, Table 1).

*Dipsacus asper* and *D. japonicus* share a globose head inflorescence; however, the corolla color of D. asper is yellowish or white (Figure 1A), while that of *D. japonicus* is pinkish or purplish (Figure 1B). Moreover, *D. asper* has pinnatisect leaves with 3–6 paired segments (Figure 1D), whereas *D. japonicus* has pinnatisect or pinnatifid leaves with 2 or 3 paired segments (Figure 1E). On the other hand, *P. umbrosa* has verticillasters bearing flowers with 2-labiate corollas (Figure 1C) and undivided orbicular-ovate to ovate-oblong cauline leaves (Figure 1F, Table 2).

### 2.2. Verification of the Identification of D. asper, D. japonicus, and P. umbrosa

**Figure 2:**
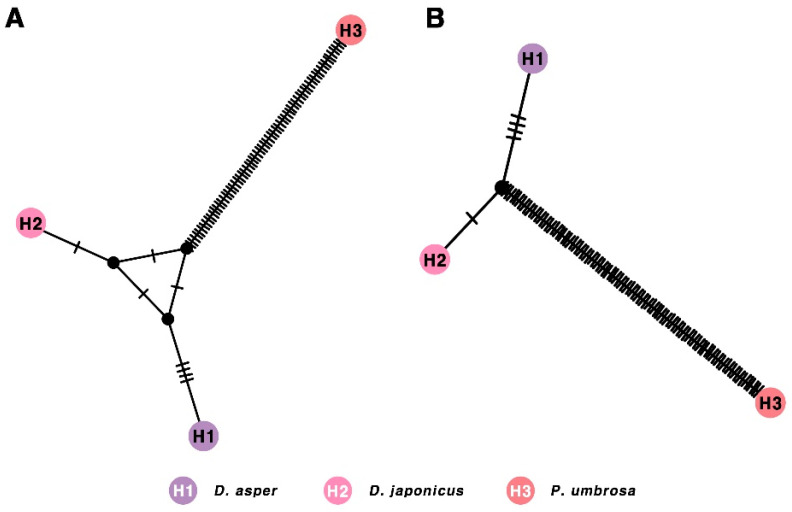
Median-Joining haplotype networks of *D. asper* (H1), *D. japonicus* (H2), and *P. umbrosa* (H3). The networks are based on the (**A**) ITS2 and (**B**) *matK* DNA sequences. Black hatch marks indicate mutations, and black dots represent inferred haplotypes.

To confirm our morphology-based identifications of *D. asper, D. japonicus*, and *P. umbrosa* we conducted DNA barcoding and haplotype network analyses on eight samples representing the three species. The analyses focused on the ITS2 and *matK* sequences, which were aligned to identify nucleotide variations (Table 3). In the ITS2 region, 77 parsimony-informative and variable sites were identified, accounting for 24.84% of the entire sequence, with a nucleotide diversity of 0.11149. The *matK* sequences exhibited 102 informative and variable sites, representing 21.88% of the sequence, with a nucleotide diversity of 0.09555. A comparison between *D. asper* and *D. japonicus* revealed seven nucleotide differences in the ITS2 sequences and five differences in the *matK* sequences. Neither of the sequences exhibited insertions or deletions (indels) between the two species. Sequence length differences were observed between *P. umbrosa* and the other two species, with the ITS2 and *matK* sequences of *D. asper* and *D. japonicus* being 5 bp and 6 bp shorter, respectively. Haplotype networks based on the ITS2 and *matK* sequences were used to visualize the genetic relationships among the three species (Figure 2). Three distinct haplotypes were identified: H1, corresponding to *D. asper*; H2, corresponding to *D. japonicus*; and H3, corresponding to *P. umbrosa*. In the ITS2 region, six mutations were identified between H1 (*D. asper*) and H2 (*D. japonicus*), 75 mutations between H1 and H3 (*P. umbrosa*), and 72 mutations between H2 and H3. Similarly, in the *matK* sequence, five mutations were detected between H1 and H2, while 101 and 98 mutations were observed between H1 and H3, and H2 and H3, respectively. Although the genetic differences between H1 and H2 were smaller compared to those between H1 and H3 or H2 and H3, all three species were clearly distinguishable based on the analyses.

### 2.3. Effects of Dipsaci Radix, Phlomidis Radix, and D. japonicus Extracts on Bone Resorption

**Figure 3:**
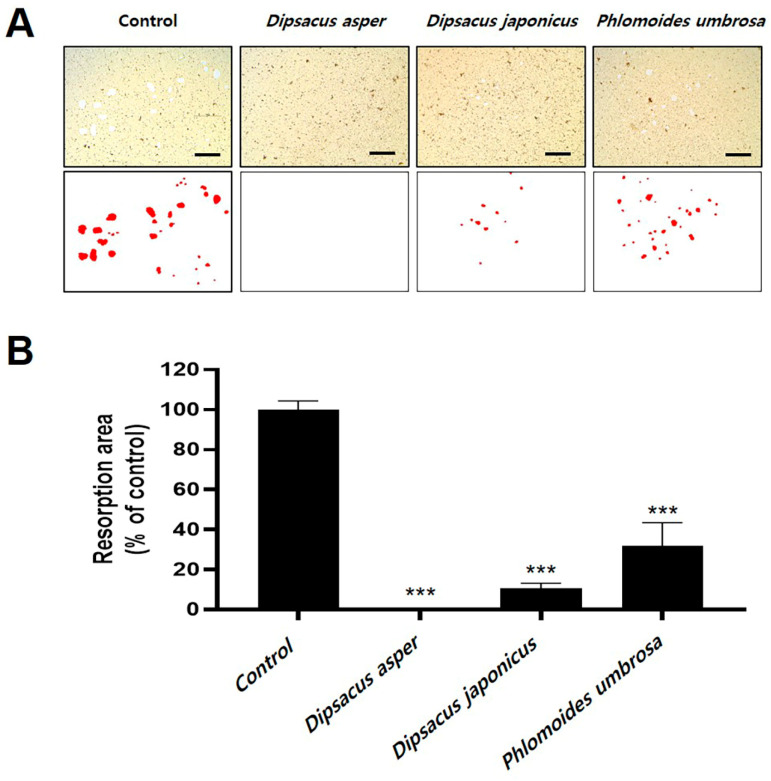
Effects of *D. asper*, *D. japonicus*, and *P. umbrosa* extracts on bone resorption by mature osteoclasts. (**A**) Hydroxypatite-adherent cells on hydroxyapatite-coated plates were imaged under a light microscope after exposure to cultures containing mature osteoclasts (control) or mature osteoclasts and one of the three extracts, all scale bars = 200 μm and (**B**) The proportion of resorbed area was quantified. Data are presented as the mean ± SD. Statistical significance levels for comparisons between the treatments and the control are represented by asterisks: *** *p* < 0.001 vs. control (DMSO).

To confirm the effect of the microsphere extracts on osteoclast bone resorption in vitro, mature osteoclasts were co-cultured with osteoblasts, and then the cultures were transferred to a plate coated with hydroxyapatite and incubated with Dipsaci Radix (*D. asper*), Phlomidis Radix (*P. umbrosa*), and *D. japonicus* extracts. In the control group treated with dimethyl sulfoxide (DMSO), many larger areas where hydroxyapatite had been absorbed by osteoclasts were observed, while in the wells containing Dipsaci Radix, Phlomidis Radix, or *D. japonicus* extract, the absorbed areas were smaller, and less hydroxyapatite appeared to have been absorbed (Figure 3A). To quantify this difference, the proportion of pit area was measured for each treatment, and a significantly reduced percentage of the hydroxyapatite absorption in the presence of all three extracts was confirmed (Figure 3B). Thus, the abilities of Dipsaci Radix, Phlomidis Radix, and *D. japonicus* extracts to inhibit osteoclast bone material absorption were confirmed through a bone resorption co-culture assay.

### 2.4. Effects of Dipsaci Radix, Phlomidis Radix, and D. japonicus Extracts on Osteoclast Formation

**Figure 4:**
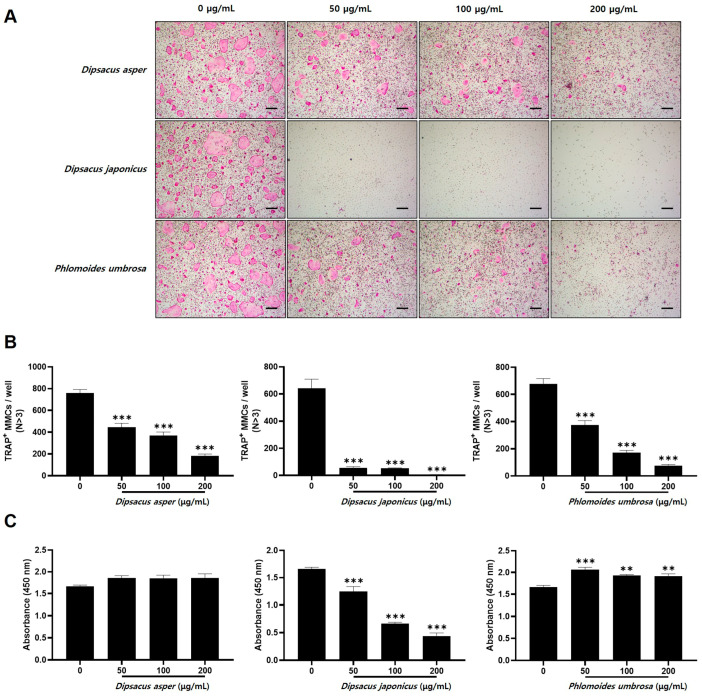
Effects on osteoclast differentiation of the ethanol extracts of *D. asper*, *D. japonicus*, and *P. umbrosa* at concentrations of 50, 100, and 200 µg/mL. (**A**) Tartrate-resistant acid phosphatase (TRAP)-positive cells photographed at 100× magnification after bone marrow macrophages were cultured with macrophage colony-stimulating factor and receptor activator of nuclear factor-κB ligand alone (control [dimethyl sulfoxide]) or in the presence of one of the three extracts. All scale bars = 200 μm. (**B**) TRAP-positive cells were counted as osteoclasts. (**C**) Cell viability was affected by the *D. asper*, *D. japonicus*, and *P. umbrosa* extracts. Statistical significance levels for comparisons between the treatments and the control are represented by asterisks: **, *p* < 0.01, and ***, *p* < 0.001 vs. control (DMSO).

To investigate the effects of Dipsaci Radix (*D. asper*), Phlomidis Radix (*P. umbrosa*), and *D. japonicus* extracts on osteoclast differentiation, phagocytes were treated with M-CSF and RANKL, essential proteins for osteoclast differentiation, and then cultured for 4 days with each extract at different concentrations. In the control group, treated with DMSO, many tartrate-resistant acid phosphatase (TRAP)-positive multinucleated osteoclasts were generated, but in the experimental groups treated with Dipsaci Radix, Phlomidis Radix, or *D. japonicus* extract, the formation of TRAP-positive osteoclasts was suppressed in a concentration-dependent manner (Figure 4A,B).

To assess whether the effects of the extracts were related to cytotoxicity, XTT experiments were performed. The *D. japonicus* extract exhibited cytotoxicity in this study, but the Dipsaci Radix and Phlomidis Radix extracts were confirmed to be non-toxic (Figure 4C).

## 3. Discussion

The medicinal and common names of *D. asper*, *D. japonicus*, and *P. umbrosa* have a complicated history. The medicinal name of the original species associated with Dipsaci Radix is the same as the common name of Phlomidis Radix: “Sok-Dan” (Table 1). Moreover, in various global plant names databases, *D. asperoides* C. Y. Cheng et T. M. Ai is treated as a heterotypic synonym of *D. asper* Wall. ex DC., and *Phlomis umbrosa* Turczaninow is treated as a heterotypic synonym of *Phlomoides umbrosa* (Turcz) Kamelin & Makhm [24,25,26,27,28]. We believe that the scientific names of the original species associated with Dipsaci Radix and Phlomidis Radix listed in the KHP should be changed to the currently accepted names used in this study (Table 1).

*Dipsacus asper* is recognized as the origin species of Dipsaci Radix in South Korea [4], China [29], and Hong Kong [30]. In contrast, *D. japonicus*, a species closely related to *D. asper*, is designated as the origin species in Vietnam [31]. Although *D. japonicus* had some side effects in this study, it also had positive effects on bone resorption and osteoclast differentiation. Notably, the processing of herbal medicines significantly affects their pharmacological and toxicological properties [32]. Therefore, further studies on the pharmacological efficacy and toxicity of *D. japonicus* are necessary to fully evaluate its potential as an additional origin species in the Korean Pharmacopoeia. *D. asper* and *D. japonicus* plants can be easily distinguished by characteristics such as inflorescence and leaf morphologies. However, it is challenging to differentiate them once they are processed into root-based medicinal materials. Therefore, identification and discrimination must rely on not only morphological and anatomical traits but also molecular markers.

Given these challenges, DNA barcoding provides a robust molecular tool for accurate species authentication and quality control [9]. Our analysis of ITS2 and *matK* sequences revealed distinct and reliable genetic differences among *D. asper*, *D. japonicus*, and *P. umbrosa*. Specifically, the ITS2 region exhibited 77 parsimony-informative sites with a nucleotide diversity of 0.11149, while the *matK* gene region contained 102 informative sites with a diversity of 0.09555. These values reflect the substantial sequence divergence between *P. umbrosa* and the two *Dipsacus* species. Although the nucleotide differences between *D. asper* and *D. japonicus* were fewer (seven sites in ITS2 and five in *matK*), they were consistent and sufficient for reliable species discrimination. The haplotype network further supported this result by identifying three distinct haplotypes corresponding to the three species. The concurrent use of both markers improves the accuracy and robustness of species authentication. These findings are consistent with previous reports highlighting the efficacy of the ITS2 and *matK* regions as reliable markers for distinguishing Dipsaci Radix from Phlomidis Radix [13,14,33], and they further demonstrate the practical applicability of DNA barcoding for the quality control of medicinal plant resources [34,35,36]. Importantly, DNA barcoding has become a crucial step in clarifying the taxonomic identities of pharmacopoeial species, ensuring that evaluations of their pharmacological efficacy and safety are based on correctly identified species.

Recent studies have shown that bone maintains normal homeostasis and repair through interactions between osteoclasts, which break down and absorb bone tissue; immune-regulatory cells; and osteoblasts, which form bone tissue [37]. This process plays a critical role in bone formation and homeostasis and is regulated by two complex processes: cell signaling and transcriptional gene expression [38]. Osteoclasts derived from hematopoietic stem cells differentiate into multinucleated cells when exposed to certain cytokines, such as M-CSF, which is necessary for the survival and proliferation of precursor monocytes/phagocytes, and RANKL, which is essential for osteoclast differentiation [39]. These cytokines initiate the differentiation of precursor cells into mature osteoclasts with bone resorption ability through various transcription factor-regulated processes [40]. Therefore, the inhibition of both osteoclast differentiation and the bone resorption function of mature osteoclasts can be considered important indicators in treating osteoporosis [41].

Previous research has confirmed the effectiveness of Phlomidis Radix in inhibiting osteoclast differentiation and bone resorption [20], and Phlomidis Radix increased bone density in mouse and rat osteoporosis models, alleviating osteoporosis symptoms measured using various indices [21,22].

In this experiment, we confirmed that Dipsaci Radix (*D. asper*), *D. japonicus*, and Phlomidis Radix (*P. umbrosa*) extracts inhibit osteoclast differentiation and prevent bone resorption using osteoclasts derived from bone marrow phagocytes using RANKL. Many bone diseases, including osteoporosis and rheumatoid arthritis, are caused by excessive osteoclastic bone resorption activity [42,43]. The present study assessed the effects of *D. japonicus* extracts on osteoclast differentiation and bone resorption without using a pharmacological reference standard [44,45]. While the extracts showed inhibitory activity relative to untreated controls, the absence of a positive control limits direct comparison with clinically established anti-resorptive agents. Future experiments incorporating bisphosphonates or other standard drugs will allow more precise evaluation of the pharmacological significance and relative potency of the extracts. Dipsaci Radix exhibited suppressive effects on bone resorption and osteoclast differentiation, while Phlomidis Radix exhibited similar but reduced effects. *D. japonicus* extract reduced osteoclast differentiation; the concentrations applied were confirmed to be within non-cytotoxic ranges. This supports the interpretation that the effect is due to targeted interference with osteoclastogenic signaling rather than cell death [46,47]. Nonetheless, further mechanistic studies are needed to distinguish specific anti-osteoclast activity from residual cytotoxic influences conclusively. On the other hand, *D. japonicus* strongly suppressed bone resorption and osteoclast differentiation, but it produced some side effects. The XTT assay was used for cytotoxicity evaluation. We recognize that polyphenols in plant extracts can interfere with tetrazolium-based measurements. Confirmation with the MTT assay and morphological assessment provided additional confidence in the observed effects. To fully delineate cytotoxicity from targeted osteoclast inhibition, future studies should include apoptosis markers and flow cytometric analyses [48,49]. *D. asper* and *P. umbrosa* have well-documented ethnomedicinal applications, the in vitro concentrations used in this study were selected primarily to balance biological activity with cellular safety. Direct comparison with traditional dosing is complicated by pharmacokinetic and metabolic differences [50]. Future studies should aim to integrate estimated human-equivalent doses and pharmacokinetic parameters to evaluate whether the observed in vitro effects correspond to clinically meaningful levels [15]. The observed side effects in this study were limited to reduced viability and morphological alterations of osteoclast precursor cells at higher extract concentrations. These in vitro findings suggest potential cytotoxicity but do not provide information on systemic or organ-specific toxicity. The extract concentrations in the present osteoclast assays were chosen to balance biological activity with cellular safety. Preliminary cytotoxicity tests established non-toxic ranges, while literature on related phytochemicals helped refine dosing. Although traditional usage provides context, in vitro concentrations were primarily determined to allow clear observation of effects on osteoclast differentiation without inducing non-specific cytotoxicity. Comparative evaluation with existing safety data or reported adverse reactions is limited due to scarce documentation. Comprehensive toxicological studies are necessary to clarify the relevance of these cellular effects to human safety and clinical application.

In Vietnam, the dried roots of *D. japonicus* are widely used to reduce inflammation and ease pain, particularly for conditions such as sore tendons, sprains, and joint pain [51,52]. Also, recent studies found that saponin XII, a compound isolated from the roots of this plant, can reduce the proliferation of acute myeloid leukemia cells [53]. In addition to finding processing methods that directly reduce the contents of toxic constituents [54], further research should be conducted to identify additional pharmacological effects.

## 4. Materials and Methods

### 4.1. Plant Materials

*Dipsacus japonicus* and *Phlomoides umbrosa* plants were collected from their natural habitats in Korea, while *D. asper* was obtained from the Agricultural Seedling Station (Aewol-eup, Jeju-si, Jeju-do, Republic of Korea). Species identification was confirmed based on descriptions of Flora of China [55,56] and Flora of Korea [57]. All samples were assigned unique identification codes and registered with the Korean Herbarium of Standard Herbal Resources (Index Herbarium code KIOM). Voucher specimens were deposited in the KIOM herbarium at the Korea Institute of Oriental Medicine (Naju, Republic of Korea). Details of these samples are listed in Table S1.

### 4.2. Morphological Observations

Images of the inflorescences and leaves of the studied species were captured using a digital camera. The terminology used for major morphological characteristics followed Li and Hedge [55], Hong et al. [56], and the Flora of Korean Editorial Committee [57,58].

### 4.3. Construction of Haplotype Network Using DNA Barcode Sequences

To assess the accuracy of our identifications of the samples from the three species, we performed a DNA barcode analysis using ITS2 and *matK* sequences. Leaves from the samples were frozen in liquid nitrogen and ground, and DNA was extracted using a modified cetyltrimethylammonium bromide (CTAB) method [59]. DNA purity and concentration were evaluated using a BioDrop uLite spectrophotometer (Biochrom Ltd., Cambridge, UK). The polymerase chain reaction (PCR) reaction mixtures contained 1 µL of DNA template (10 ng), 2 µL each of forward and reverse primers (10 pmol), 10 µL of Taq polymerase (Solg™ 2X PCR Smart mix, Solgent, Daejeon, Korea), and 7 µL of double-distilled water, making a total volume of 20 µL. *MatK* primers were designed from conserved regions of *Dipsacus* and *Phlomoides* sequences obtained from the NCBI database (https://www.ncbi.nlm.nih.gov/, accessed on 30 August 2025). Primer design was performed using Primer-BLAST [60] (Tables S1 and S2). Amplifications were per-formed using an Eppendorf Mastercycler system (Eppendorf, Hamburg, Germany) with the following program: initial denaturation at 95 °C for 2 min, 35 cycles of denaturation at 95 °C (50 s), annealing at 54 °C (ITS2) or 58 °C (*matK*) for 50 s, extension at 72 °C for 50 s, and final extension at 72 °C for 5 min. PCR products were separated on 2% agarose gels at 150 V for 40 min, excised from the gels, and sent to Bionics (Seoul, Korea) for Sanger sequencing. The generated sequences were quality-checked in Geneious Prime (v.2024.0.7), aligned using MAFFT (v.7.388) [61], and compared with reference sequences downloaded from the NCBI database to confirm species identification. The aligned sequences were further visualized in BioEdit (v.7.2.5) [62] (Figure S1). Parsimony-informative and variable sites were identified using MEGA (v. 11) [63]. Nucleotide diversity, indels, and haplotypes were analyzed with DnaSP (v.6) [64], and haplotype networks were constructed using the median-joining method in PopArt (v.1.7) [65].

### 4.4. Isolation of Bone Marrow Cells

To isolate bone marrow cells, five-week-old male institute of cancer research (ICR) mice were euthanized, and their femurs and tibias were aseptically removed and stripped of surrounding tissues. The ends of the long bones were cut off, and the bone marrow was flushed out using a 1 mL syringe to collect the bone marrow cells. The isolated cells were cultured for one day in α-minimum essential medium (MEM) supplemented at 10% with fetal bovine serum (FBS) and at 1% with penicillin/streptomycin. Non-adherent cells were then collected and cultured in α-MEM containing 10% FBS, 1% penicillin/streptomycin, 30 ng/mL of macrophage colony-stimulating factor (M-CSF) for 3 days to allow differentiation into macrophage precursors. Then, the adherent macrophages were used for further experiments. For osteoclast differentiation, the macrophages were treated with a 30 ng/mL M-CSF, 100 ng/mL receptor activator of nuclear factor-κB ligand (RANKL) solution in the presence of *D. asper, D. japonicus*, or *P. umbrosa* root extract each at concentrations of 25, 50, and 100 µg/mL. After 3 days, the medium was replaced with the same medium, and the following day, cells were stained with TRAP solution. The TRAP-positive (red-stained) cells were counted to assess osteoclast differentiation [66].

### 4.5. Cytotoxicity Assay

Macrophages were seeded at a density of 1 × 10^4^ cells/well in a 96-well plate and treated in a 30 ng/mL M-CSF solution containing varying concentrations of *D. asper, D. japonicus*, or *P. umbrosa* root extract for 3 days. After incubation, 50 µL of XTT solution was added to each well, and the plates were incubated for an additional 4 h. Absorbance was then measured at 450 nm using an ELISA reader (Molecular Devices, San Jose, CA, USA) to determine cell viability [67].

### 4.6. Bone Resorption Assay

Male ICR mice (4 weeks old) were sourced from Doo Yeol Biotech (Seoul, Republic of Korea) and acclimated for 7 days prior to testing. Animals were maintained in controlled environmental parameters (23 ± 2 °C, 50 ± 5% humidity) with a 12 h light/dark cycle (08:00–20:00) and ventilated at 13–18 air exchanges hourly. Standard rodent feed and water were provided ad libitum. All protocols were authorized following review by the Chonnam National University Institutional Animal Care and Use Committee (CNU IACUC-YB-2025-82), adhering to the NIH Guide for the Care and Use of Laboratory Animals and Korean animal welfare legislation. To obtain mature osteoclasts, bone marrow cells from the femurs and tibias of 5-week-old ICR mice and calvarial osteoblasts from 1-day-old ICR mice were co-cultured in collagen-coated 90 mm culture dishes in the presence of 1α,25-dihydroxyvitamin D_3_ (VitD_3_) and prostaglandin E_2_ for 6 days. After co-culturing, cells were detached using 0.1% collagenase and seeded into hydroxyapatite-coated 96-well plates. *D. asper, D. japonicus*, or *P. umbrosa* root extract was added at a concentration of 200 µg/mL, and the cells were incubated for 12 h. Then, cells were washed with distilled water and observed under an optical microscope. The resorbed area of hydroxyapatite was quantified using Image Pro-Plus software v. 4.0 (Media Cybernetics, Rockville, MA, USA) [68].

### 4.7. Statistical Analysis

TRAP-positive multinucleated cells were quantified from three independent experiments (*n* = 3), each performed in triplicate. Bone resorption assays were also carried out in three independent experiments (*n* = 3). Data are presented as mean ± standard deviation (SD). Statistical analysis was performed using one-way ANOVA followed by Tukey’s post hoc test, which accounts for multiple comparisons.

## 5. Conclusions

This study provides comprehensive data on the nomenclature, morphological characteristics, DNA barcode sequences, and biological activities of three species of medicinal plants: *D. asper*, *D. japonicus*, and *P. umbrosa*. We detailed important morphological differences between the whole plants and proposed corrections to the scientific names used for these plants in the KHP. Further, this study provided scientific evidence for the previously known effect of Dipsaci Radix on bone tissue and also demonstrated that caution is necessary when using Phlomidis Radix and the close relative of *D. asper*, *D. japonicus*, both of which can be misused. Consequently, it is considered essential to conduct more in-depth research into the mechanism of the effects of Dipsaci Radix, Phlomidis Radix, and *D. japonicus*, as well as into component analysis.

## Figures and Tables

**Table 1:** Comparative list of the medicinal materials used in the study and their corresponding scientific and Korean names, as registered in the Korean Herbal Pharmacopoeia (KHP) and Flora of Korea.

Pharmacopoeia (KHP)				Flora and Plant Taxonomy (Flora of Korea)	
Medicinal name	Scientific name	Medicine name	Species name	Scientific name	Korean common name
Dipsaci Radix	*Dipsacus asperoides* C. Y. Cheng et T. M. Ai	Sok-Dan	Cheon-Sok-Dan	*Dipsacus asper* Wall. ex DC.	None (not native to Korea)
Adulterant of Dipsaci Radix	Not listed	Not listed	Il-Bon-Sok-Dan	*Dipsacus japonicus* Miq.	San-To-Kki-Kkot
Phlomidis Radix	*Phlomis umbrosa* Turczaninow	Han-Sok-Dan	Han-Sok-Dan	*Phlomoides umbrosa* (Turcz.) Kamelin & Makhm.	Sok-Dan

**Table 2:** Major morphological characteristics of *D. asper*, *D. japonicus*, and *P. umbrosa*.

Characteristic	*D. asper*	*D. japonicus*	*P. umbrosa*
Height	ca. 2 m	ca. 1.5 m	ca. 1.5 m
Root	Taproots, fleshy	Taproots, not fleshy	Taproots, lateral tuberous
Stem	Ridged, with sparse recurved spines	Ridged, with sparsely recurved spines	4-angled, with moderate to dense recurved hairs
Leaf type	Basal and cauline, petiolate	Basal and cauline, petiolate	Cauline, petiolate
Leaf shape	Elliptic, pinnatisect, 3–6 paired segments	Elliptic-ovate to elliptic, pinnatisect or pinnatifid, 2 or 3 paired segments	Orbicular-ovate to ovate-oblong, not divided
Leaf apex	Acute or acuminate	Acute	Acute to acuminate
Leaf margins	Sparsely serrate	Sparsely serrate	Serrate-dentate to irregularly crenate
Inflorescence shape	Globose head	Globose head	Verticillaster
Inflorescence position	Terminal	Terminal	Axils of floral leaves
No. of flowers per inflorescence	Many	Many	2–8-flowered
Corolla color	Yellowish or white	Pinkish or purplish	Purplish red
Corolla shape	Funnelform	Funnelform	2-labiate
Stamens	Exserted	Slightly exserted	Included
Anther color	Purplish or black	Black	Black
Fruits	Achenes	Achenes	Nutlets

**Table 3:** Statistics from the DNA barcode analyses of *D. asper*, *D. japonicus*, and *P. umbrosa*.

Species	DNA	Alignment Length (bp)	Parsimony Informative Sites		Variable Sites		Nucleotide Diversity (Pi)	No. of Indels	No. of Haplotypes
			No.	%	No.	%			
*D. asper vs. D. japonicus vs. P. umbrosa*	ITS2	310 bp	77	24.84%	77	24.84%	0.11149	14	3
	*matK*	465 bp	102	21.88%	102	21.88%	0.09555	6	3
*D. asper vs. D. japonicus*	ITS2	305 bp	7	2.30%	7	2.30%	0.01224	0	2
	*matK*	459 bp	5	1.09%	5	1.09%	0.00581	0	2

## Data Availability

The ITS2 (GenBank accession number: PQ866042–PQ866047) and *matK* (PQ868268–PQ868273) sequences generated in this study have been deposited in the NCBI database (https://www.ncbi.nlm.nih.gov/, accessed on 10 August 2025).

## Supplementary Materials

The following supporting information can be downloaded at https://www.mdpi.com/article/10.3390/ph18091418/s1: Figure S1: Sequence alignment used for DNA barcoding illustrating nucleotide differences; Table S1: Details of the *D. asper*, *D. japonicus*, and *P. umbrosa* samples used in the DNA barcode analysis; Table S2: Information on the primers used in the DNA barcode analysis.
