# Supplementary material for: Integrative Study of Dipsaci Radix and Phlomidis Radix: Nomenclature, Morphology, DNA-Based Authentication, and Comparative Effects on Osteoclastogenesis
Source: Pharmaceuticals (Basel). 2025 Sep 20;18(9):1418. doi: 10.3390/ph18091418 (PMC12472938; doi:10.3390/ph18091418)
Supplement: Supplementary file 1 [file pharmaceuticals-18-01418-s001.zip › pharmaceuticals-3853536-supplementary.pdf]

Supplementary Materials

Integrative Study of Dipsaci Radix and Phlomidis Radix: Nomenclature, Morphology, DNA-Based Authentication, and Comparative Effects on Osteoclastogenesis

Jun-Ho Song [1,†], Yun-Soo Seo [2,†], Yeseul Kim [3], Sohee Jeong [4], Sungyu Yang [2], Goya Choi [2], Joong-Sun Kim [4,*] and Inkyu Park [3,*]

[1] Department of Biology, Chungbuk National University, Cheongju 28644, Republic of Korea; jhsong@chungbuk.ac.kr

[2] Herbal Medicine Resources Research Center, Korea Institute of Oriental Medicine, Naju, 58245, Republic of Korea; sys0109@kiom.re.kr (Y.-S.S.); sgyang81@kiom.re.kr (S.Y.); serparas@kiom.re.kr (G.C.)

[3] Department of Biology, Changwon National University, Changwon 51140, Korea; 20257520@gs.cwnu.ac.kr

[4] College of Veterinary Medicine and BK21 FOUR Program, Chonnam National University, Gwangju 61186, Republic of Korea; sohee0460@naver.com

* Correspondence: centraline@jnu.ac.kr (J.-S.K.); pik6885@cwnu.ac.kr (I.P.); Tel.: +82-62-530-2815 (J.-S.K.); +82-55-213-3455 (I.P.).

† These authors contributed equally to this work.

Table S1. Details of the *D. asper*, *D. japonicus*, and *P. umbrosa* samples used in the DNA barcode analysis.

No.	Species	Voucher number	Collection information	Morphological analysis	DNA analysis	Pharmacological analysis
1	*Dipsacus asper*	KIOM201201004804	Agricultural Seedling Station, Aewol-eup, Jeju-si, Jeju-do, Korea	o	o	o
2		KIOM201701018837	Goejeong-ri, Cheongju-si, Chungcheongbuk-do, Korea	o	o	
3	*Dipsacus japonicus*	KIOM201801020871	Sobaeksan Mt., Yeongju-si, Gyeongsangbuk-do, Korea	o	o	o
4		KIOM201701018778	Yeongchun-myeon, Danyang-gun, Chungcheongbuk-do, Korea	o	o	
5		KIOM201701018779	Yeongchun-myeon, Danyang-gun, Chungcheongbuk-do, Korea	o	o	
6	*Phlomis umbrosa*	KIOM201401008313	Gadeoksan Mt., Sangsami-dong, Taebaek-si, Gangwon-do, Korea	o	o	o
7		KIOM201401010021	Dundeoksan Mt., Wanjang-ri, Gaeun-eup, Mungyeong-si, Gyeongsangbuk-do, Korea	o	o	
8		KIOM201401011171	Seondalsan Mt., Ojeon-ri, Murya-myeon, Bonghwa-gun, Gyeongsangbuk-do, Korea	o	o	

Table S2. Information on the primers used in the DNA barcode analysis.

Primer name	Primer sequence (5'→3')
ITS2 F	ATGCGATACTTGGTGTGAAT
ITS2 R	GACGCTTCTCCAGACTACAAT
matK_Phlomis F	TTCGTCTTTCTCCGTAACCA
matK_Phlomis R	ATCCGCCCAAACTACCTTAC
matK_Dipsacus F	CAAAGAAAGCCAGTTCCCCT
matK_Dipsacus R	ACGCCCAAATCGGTCAATAA

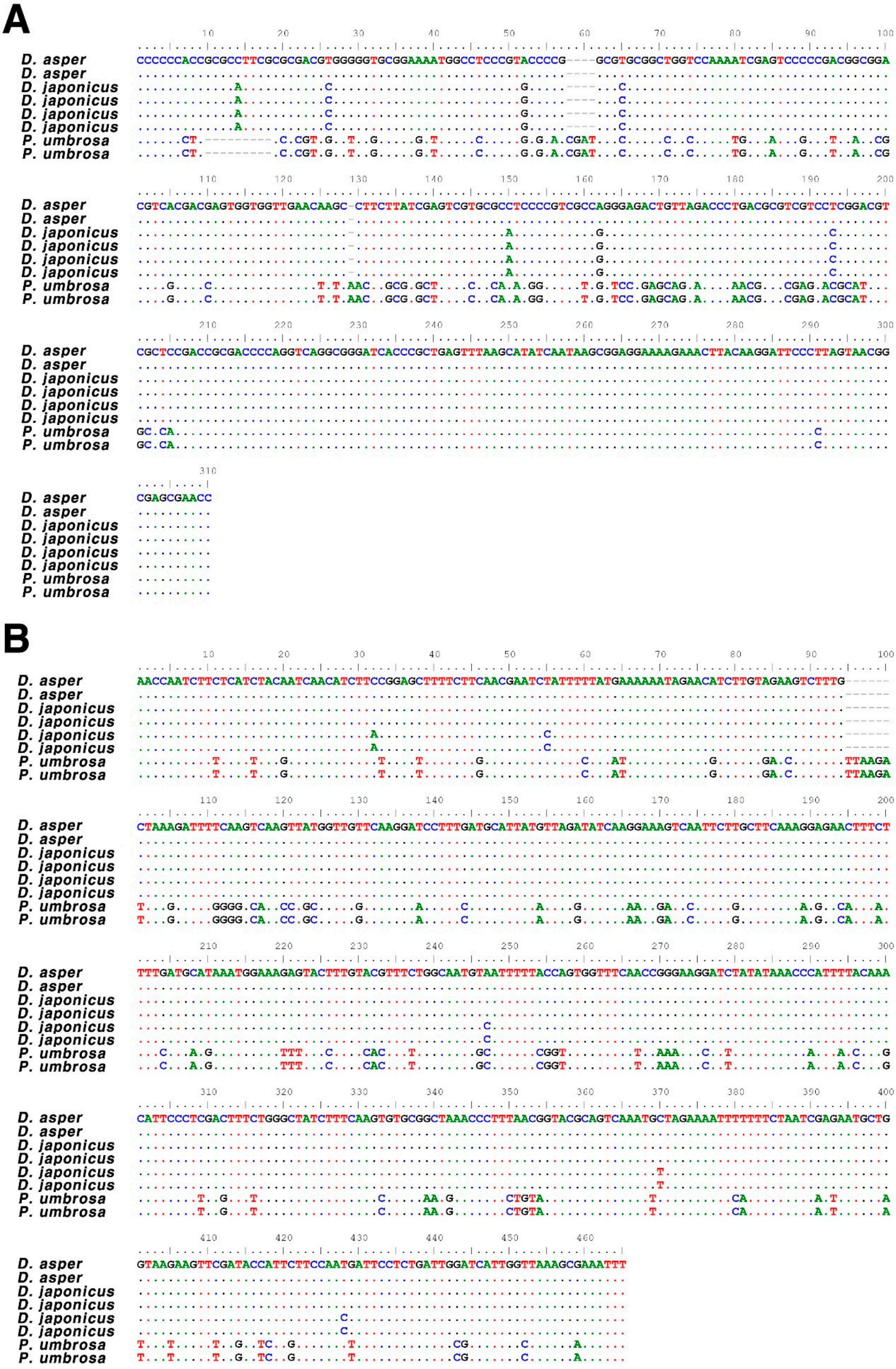

Figure S1. Sequence alignment used for DNA barcoding illustrating nucleotide differences.
